# T-cell receptor signal strength and epigenetic control of Bim predict memory CD8^+^ T-cell fate

**DOI:** 10.1038/s41418-019-0410-x

**Published:** 2019-09-26

**Authors:** Kun-Po Li, Brian H. Ladle, Sema Kurtulus, Allyson Sholl, Sharmila Shanmuganad, David A. Hildeman

**Affiliations:** 1 Immunology Graduate Program, Cincinnati Children’s Hospital Medical Center and the University of Cincinnati College of Medicine, Cincinnati, OH 45229 USA; 2 Cancer and Blood Diseases Institute, Cincinnati Children’s Hospital Medical Center and the University of Cincinnati College of Medicine, Cincinnati, OH 45229 USA; 3 Division of Immunobiology, Cincinnati Children’s Hospital Medical Center and the University of Cincinnati College of Medicine, Cincinnati, OH 45229 USA; 4 Present Address: Johns Hopkins Hospital, 1800 Orleans Street, The Charlotte R. Bloomberg Children’s Center Building, 11th Floor, Baltimore, MD 21287 USA

**Keywords:** Immune cell death, T cells

## Abstract

Most effector CD8^+^ T cells die, while some persist and become either “effector” (T_EM_) or “central” (T_CM_) memory T cells. Paradoxically, effector CD8^+^ T cells with greater memory potential have higher levels of the pro-apoptotic molecule Bim. Here, we report, using a novel Bim-mCherry knock-in mouse, that cells with high levels of Bim preferentially develop into T_CM_ cells. Bim levels remained stable and were regulated by DNA methylation at the Bim promoter. Notably, high levels of Bcl-2 were required for Bim^hi^ cells to survive. Using Nur77-GFP mice as an indicator of TCR signal strength, Nur77 levels correlated with Bim expression and Nur77^hi^ cells also selectively developed into T_CM_ cells. Altogether, these data show that Bim levels and TCR signal strength are predictive of T_EM_- vs. T_CM_-cell fate. Further, given the many other biologic functions of Bim, these mice will have broad utility beyond CD8^+^ T-cell fate.

## Introduction

CD8^+^ T cells play a critical role in eliminating intracellular pathogen infected cells or tumor cells. During primary activation, antigen-specific CD8^+^ T cells expand and perform effector functions, such as cytotoxic killing and producing pro-inflammatory cytokines. After the antigen is eliminated, most of the CD8^+^ T cells die via apoptosis during the immune contraction phase, while a few remaining CD8^+^ T cells survive and establish immune memory [1–3]. Mechanisms underlying this cell fate choice are unclear and remain an active focus of investigation in many labs.

Previous studies showed that precursors of CD8^+^ memory T cells can be identified at the early stage of immune response [4–6]. These pre-memory cells express a KLRG1^lo^CD127^hi^ cell surface phenotype and employ a distinct transcriptional program from their counterpart, KLRG1^hi^CD127^lo^ terminal effector cells [5, 7]. Adoptive transfer studies showed that both central memory T cells (T_CM_) and effector memory T cells (T_EM_) can emerge from the pre-memory pool. Functionally, in response to a secondary challenge, T_CM_ cells exhibit better ability to expand while T_EM_ cells rapidly perform cytotoxic functions to eliminate target cells [8–10].

Following the expansion and differentiation of CD8^+^ T cells, the majority of terminal effector cells die while some pre-memory cells survive. We and others have shown that the BH3-only, pro-apoptotic Bcl-2 family member, Bim (*Bcl2l11*) is critical for the apoptotic contraction of the response [2, 11–15]. Indeed, in the absence of Bim, the numbers of both terminal effector and pre-memory T cells are significantly increased [16]. Paradoxically, at the peak of the response, Bim levels are actually increased to a greater degree in pre-memory CD8^+^ T cells, the cells that are destined to become memory cells, relative to terminal effector cells [15]. These pre-memory cells also express higher levels of Bcl-2, which is essential to combat Bim and promote their survival [15]. Interestingly, similar to their pattern of expression in pre-memory and terminal effector cells, Bim and Bcl-2 are highly expressed in T_CM_ cells, but lowly expressed in T_EM_ cells [15]. As both T_CM_ and T_EM_ cells emerge from the high Bim-expressing pre-memory cells, it remains unclear how Bim levels are regulated as cells transition from the effector to memory stage.

*Bcl2l11* gene expression has been reported to be controlled by transcriptional, post-transcriptional, and post-translational mechanisms [11]. In T cells, TCR stimulation increases *Bcl2l11* messenger RNA (mRNA) and Bim protein [17–19]. Further, the magnitude of TCR stimulation has been proposed to control development of long-lived memory T cells [20]. In this regard, the Hogquist group has generated Nur77-reporter mice that express GFP, downstream of the Nur77 promoter [21]. GFP expression in these mice correlated with the degree of TCR signal strength and was not affected by non-TCR signals such as cytokines or co-stimulatory molecules. Thus, these mice are an excellent model to examine the relationship between TCR signal strength, Bim expression, and memory development. Unfortunately, Bim is an intracellular protein making it impossible to manipulate cells on the basis of Bim expression and maintain cell viability. Therefore, to track the expression of Bim and retain cell viability, we generated Bim-mCherry-reporter mice in which we inserted an internal ribosome entry site (IRES)-mCherry cassette into the 3ʹ-untranslated region (UTR) of the *Bcl2l11* gene. We used these mice to interrogate the expression of Bim across an antiviral T-cell response from effector to memory development. In addition, we crossed the Bim-mCherry mice to Nur77-GFP reporter mice. Our data show that Bim expression is associated with TCR signal strength and both can predict T_EM_- vs. T_CM_-cell fate. These data have significant implications for our understanding of memory T-cell development.

## Results

### Generation of Bim-mCherry reporter mice

Our and others’ prior work show that Bim is critical for contraction of T-cell responses [12–16, 22, 23]. Subsequently, we made the paradoxical observation that the levels of Bim are actually higher in the CD8^+^ T cells that are destined to be long-lived memory T cells [15]. That observation suggested that Bim levels might predict memory T-cell fate. Unfortunately, as Bim is an intracellular molecule, sorting cells based on Bim levels while maintaining cell viability was impractical. To overcome this obstacle, we generated Bim-mCherry reporter mice, by inserting an IRES-mCherry cDNA cassette into the 3ʹ-UTR of the *Bcl2l11* gene (Supplementary Fig. 1).

**Fig. 1:**
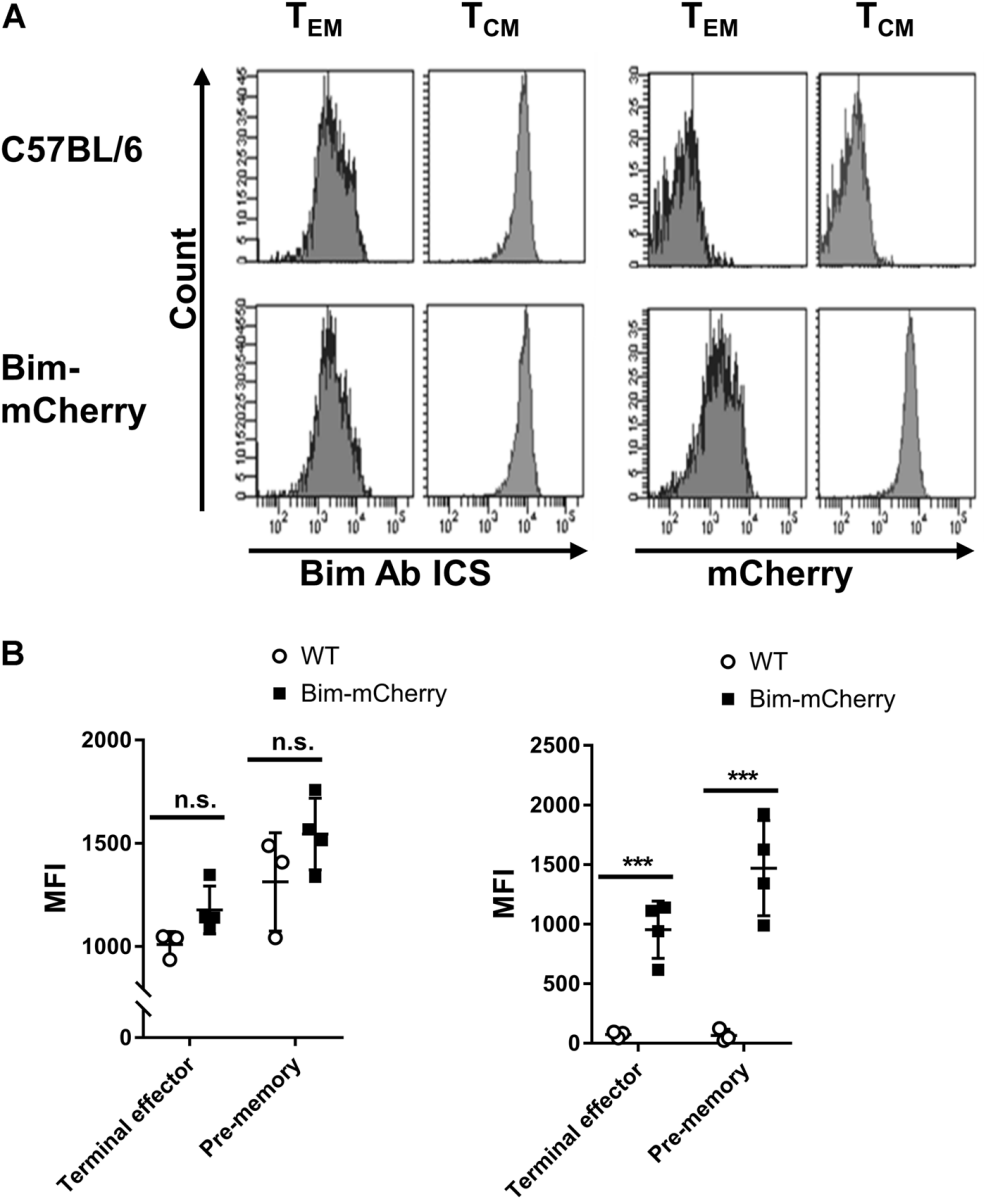
Bim-mCherry mice faithfully report Bim expression levels. **a** Representative histograms of Bim ICS and mCherry fluorescent intensities gated on C57BL/6 or Bim-mCherry reporter endogenous T_EM_ or T_CM_ cells. Plots are representative of three mice per group and repeated in two independent experiments. **b** Bim ICS and mCherry fluorescent MFI levels. Bar graphs compare LCMV-specific CD8^+^ terminal effector or pre-memory cells from LCMV-infected C57BL/6 (white bars, *n* = 3) or Bim-mCherry reporter (black bars, *n* = 4) mice measured on 10-day-post-infection (dpi). Results are representative of three independent experiments (mean ± SD, ***p < 0.001, unpaired two-tailed Student’s *t-*test)

To determine whether mCherry fluorescence faithfully reported Bim expression, we used flow cytometry to measure mCherry fluorescence and compared that to the levels of Bim measured by intracellular staining (ICS) [24] in populations of T cells that have divergent expression of Bim [15]. First, endogenous CD8^+^ T_CM_ cells had higher levels of Bim than CD8^+^ T_EM_ cells as assessed by ICS in both C57BL/6 and Bim-mCherry strains (Fig. 1a), demonstrating that the insertion of the reporter cassette did not affect Bim expression. In Bim-mCherry mice, mCherry levels were higher in T_CM_ cells relative to T_EM_ cells, similar to endogenous Bim levels in control mice (Fig. 1a). Next, we infected Bim-mCherry mice with lymphocytic choriomeningitis virus (LCMV) and tracked Bim levels in LCMV-specific CD8^+^ T cells. Similar to endogenous CD8^+^ T-cell memory subsets, Bim-antibody staining of effector CD8^+^ T cells was similar in viral-specific CD8^+^ T cells from both C57BL/6 and Bim-mCherry mice (Fig. 1b). Further, mCherry fluorescence faithfully reported the levels of Bim as assessed by ICS in both MHC tetramer-defined effector subsets (Fig. 1b). Altogether, these results show that the mCherry reporter reflected Bim protein levels with high fidelity, and the insertion of the reporter cassette into the Bim locus did not affect Bim expression.

Next, we tested whether the insertion of mCherry reporter affected the function of Bim in vivo. Bim is critical for (i) restricting the development of agonist selected T cells in the thymus [25–28], (ii) overall numbers of peripheral T cells, (iii) contraction of T-cell responses [14, 16], and (iv) controlling the size of the pre-memory effector T-cell subset [16] and the T_CM_ population (Supplementary Fig. 2). We found that thymocyte development, especially the accumulation of CD4^-^CD8^-^ double-negative(DN) thymocytes, which are strictly controlled by Bim [25, 29], appeared normal (Supplementary Fig. 3A). Splenic T-cell populations were normal in the reporter mice (Supplementary Fig. 3B). Further, after LCMV infection, there was no difference in the contraction of LCMV-specific CD8^+^ T-cell responses between C57BL/6 and Bim-mCherry mice (Supplementary Fig. 3C, D). These data show that Bim function is normal in Bim-mCherry mice.

### High levels of Bim at the early stage of viral infection predict the memory fate of CD8^+^ T cells

We next crossed the Bim-mCherry mice to P14 TCR-Tg mice (whose TCR is specific to LCMV GP_33–41_ peptide presented by the class I MHC molecule, D^b^) on BoyJ (CD45.1^+^ congenic) background to test whether the levels of Bim were predictive of CD8^+^ memory T-cell fate. CD8^+^ T cells from uninfected P14-Bim-mCherry mice were transferred into C57BL/6 recipients and subsequently infected. Ten days later, P14 cells from the primary recipients were sorted for Bim^hi^ (top 25% highest mCherry expression) or Bim^lo^ (bottom 25% lowest mCherry expression) cells and transferred separately into timed-infected secondary recipients (Supplementary Fig. 4A). The Bim^hi^ and Bim^lo^ populations on day 10 post-infection had similar frequencies of effector populations defined by KLRG1 and CD127, and both lacked expression of CD62L (Supplementary Fig. 4B–D). After 14 days (day 24 post-infection), the donor cells were isolated and analyzed by flow cytometry.

**Fig. 2:**
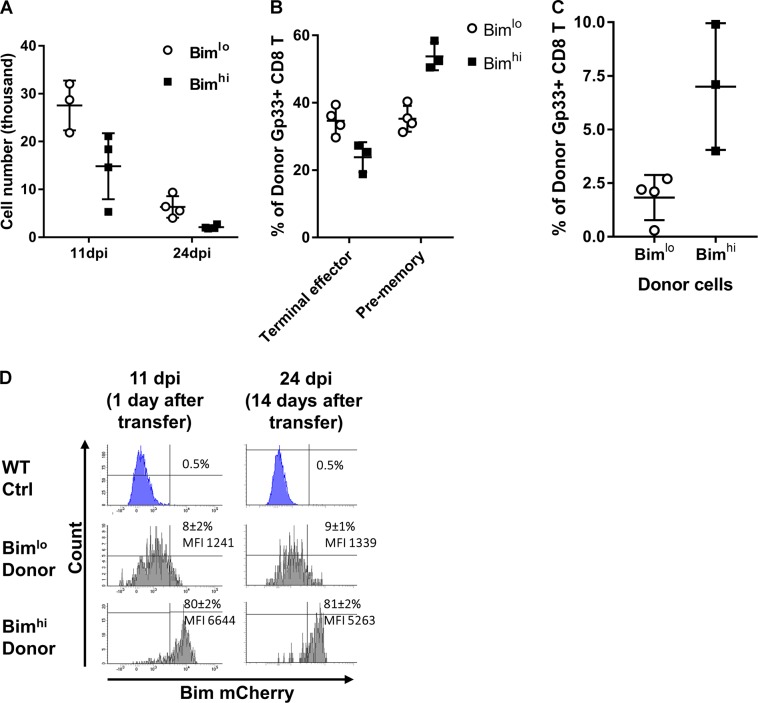
Bim^hi^ cells bias to pre-memory cells, promote T_CM_ cells and maintain Bim levels. **a** Numbers of transferred P14-Bim-mCherry reporter cells harvested on 11 dpi (1 day after transfer, *n* = 4) or 24 dpi (14 days after transfer, *n* = 4). Bar graph (mean ± SD) compares mice receiving Bim-mCherry^hi^ cells (black bars) or mice receiving Bim-mCherry^lo^ cells (white bars). Cell number fold differences are shown above horizontal lines. **b** Bar graphs (mean ± SD) show frequencies of terminal effector cells, pre-memory cells, or **c** T_CM_ cells among transferred Bim^hi^ (black bars) or Bim^lo^ (white bars) P14 cells on 24 dpi (*n* = 4). **d** Representative histograms of Bim-mCherry fluorescent intensities gated on transferred Bim^hi^ or Bim^lo^ cells harvested on 11 dpi (*n* = 3) or 24 dpi (*n* = 4). Bim^hi^ frequencies and MFI (mean ± SD) are shown. All experiments were performed twice with similar results. For all panels, *p < 0.05 (unpaired two-tailed Student’s *t*-test)

Interestingly, Bim^hi^ cells contracted more substantially than Bim^lo^ cells in recipients as lower numbers of Bim^hi^ cells were recovered from recipients compared to Bim^lo^ cells (Fig. 2a). Strikingly, at 14 days after transfer, we found that Bim^hi^ donor CD8^+^ T cells had a larger pre-memory compartment, and a smaller terminal effector compartment, relative to Bim^lo^ donor cells (Fig. 2b). In addition, a higher frequency of donor cells from the Bim^hi^ group had a central memory phenotype, displaying increased expression of CD62L (Fig. 2c). Moreover, we found that each group of CD8^+^ T cells preferentially maintained their level of Bim expression; Bim^hi^ cells remained Bim^hi^ while Bim^lo^ cells remained Bim^lo^ 14 days after transfer (Fig. 2d). The same differentiation pattern and maintenance of Bim expression was observed in independent experiments with transfer of non-TCR-transgenic CD8^+^ T cells with mCherry reporter (data not shown). These data show that Bim levels at the early stage of CD8^+^ T-cell responses are stably maintained and are predictive of memory cell fate.

### Expression levels of Bim are negatively correlated with DNA methylation patterns on Bim promoter

**Fig. 3:**
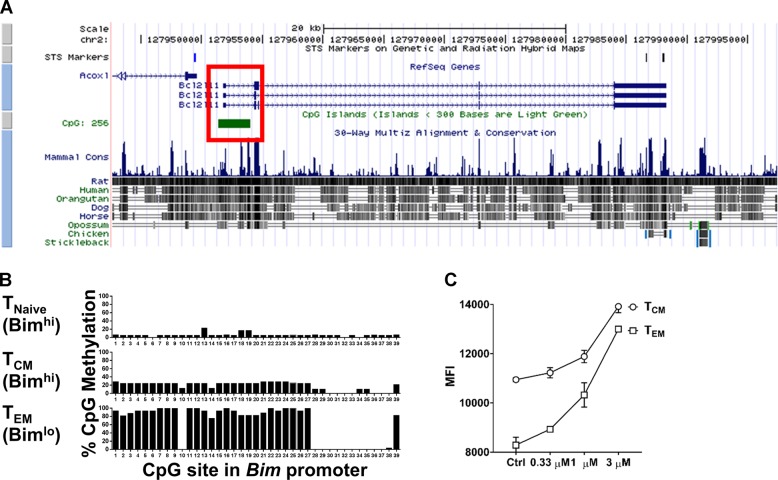
DNA methylation represses Bim expression. **a** A CpG condensed region, CpG island 256, covering the promoter, transcription start site, and 5ʹ-UTR of *Bcl2l11* gene was identified. **b** Frequencies of methylated CpG sites in the CpG island 256. Endogenous T_CM_ or T_EM_ cells from uninfected C57BL/6 mice were sorted, and the promoter region (−461/ + 4) was analyzed by bisulfite sequencing. Twenty clones from each population were tested. **c** MFI of Bim ICS from splenic CD8^+^ T cells in vitro cultured with 5-aza-2’-deoxycytidine and IL-2 (20 ng/ml) for 3 days. (Mean ± SD, *p < 0.05, **p < 0.01, comparing to untreated Ctrl. Unpaired two-tailed Student’s *t-*test). Results are representative of two independent experiments

Given the stability of Bim expression, we hypothesized that DNA methylation, an epigenetic modification repressing gene expression, may be involved in the differential control of Bim expression in CD8^+^ T cells. We first searched for potential target regions for DNA methylation, cytosine-guanine dinucleotide (CpG) condensed regions, around the *Bcl2l11* gene. From the UCSC Genome Browser database [30], we identified a conserved CpG condensed region, CpG island 256, which covers the promoter, transcription start site, and 5ʹ-UTR of the *Bcl2l11* gene (Fig. 3a). Next, we focused on the region, which begins from 461 bases before the Bim transcriptional start site to four bases after the start site (−461/ + 4), which covers the first 39 CpG dinucleotides. To determine whether CD8^+^ T cells with different levels of Bim have distinct DNA methylation patterns, we sorted endogenous T_CM_ or T_EM_ cells from uninfected C57BL/6 mice because of their differential expression of Bim (Fig. 1a). Using bisulfite sequencing, we found that the CpG sites of T_EM_ cells were highly methylated compared to T_CM_ cells, which express higher levels of Bim and have low levels of DNA methylation (Fig. 3b). Further, culture of CD8^+^ T cells with a DNA methylation inhibitor, 5-aza-2’-deoxycytidine (5-aza-dC), resulted in substantially upregulated Bim expression levels in T_EM_ cells and partially increased Bim levels in T_CM_ cells (Fig. 3c). Altogether, these data show that DNA methylation contributes to differential Bim expression in CD8^+^ T_EM_ cells.

### Bim levels and memory fate are correlated with strong TCR avidity and Nur77 expression

**Fig. 4:**
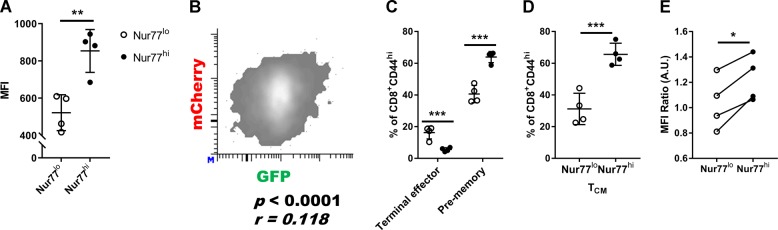
Nur77^hi^ cells have high levels of Bim, high TCR affinity, and bias to pre-memory cell and T_CM_ differentiation. **a** Splenic CD8^+^ T cells were harvested from Bim^mCherry^Nur77^GFP^ double-reporter mice with LCMV infection on day 10. H-2D^b^-GP33 tetramer^+^ CD8^+^ T cells were analyzed by flow cytometry. The Bim^mCherry^ MFI of CD8^+^ T cells with the top or the bottom 10% Nur77^GFP^ expression are shown (mean ± SD, ** *p* < 0.01, paired two-tailed Student’s *t*-test). **b** The correlation between mCherry and GFP MFI are shown in Pearson *r* and *p*-value. The flow plot is representative of four mice and repeated in two independent experiments. **c** Bar graphs (mean ± SD) show frequencies of terminal effector cells, pre-memory cells, or **d** T_CM_ cells among transferred Nur77^hi^ (black bars) or Nur77^lo^ (white bars) CD8^+^ cells on 24 dpi (*n* = 4, ***p < 0.001 unpaired two-tailed Student’s *t*-test). **e** Splenic CD8^+^ T cells were harvested from Nur77^GFP^ mice with LCMV infection on day 10. H-2D^b^-GP33 tetramer^+^ CD8^+^ T cells were stained with TCRβ Ab. The MFI ratio (tetramer/TCRβ) of cells with the top or the bottom 10% Nur77^GFP^ MFI were calculated and shown. Results are representative of two independent experiments. (*p < 0.05, paired two-tailed Student’s *t*-test)

Prior work showed that TCR stimulation increases Bim expression [17–19]. In addition, the strength of TCR stimulation has been shown to be an important factor in determining CD8^+^ T-cell memory development, although the data along these lines are controversial. Therefore, we next examined whether Bim expression and memory generation are associated with the strength of TCR stimulation. Here, we took advantage of Nur77-GFP mice, whose GFP levels are proportional to the level of TCR stimulation [21]. We first determined whether the levels of Bim were correlated with the level of Nur77 in LCMV-specific CD8^+^ T cells by generating Nur77^GFP^Bim^mCherry^ double-reporter mice. Strikingly, we observed that the LCMV-specific Nur77-GFP^hi^ CD8^+^ T cells express higher levels of Bim (Fig. 4a) and that the expression of mCherry and GFP were positively correlated (Fig. 4b). Next, we sorted the top and bottom 25% of GFP expressing T cells from Nur77-GFP mice on day 10 post-infection, which have similar frequencies of effector populations defined by KLRG1 and CD127 (Supplementary Fig. 4E), and adoptively transferred them into timed-infected recipients. Fourteen days later, recipients were sacrificed and donor LCMV-specific CD8^+^ T cells were characterized in the spleen. More pre-memory cells emerged from adoptive transferred Nur77-GFP^hi^ cells compared to Nur77-GFP^lo^ cells (Fig. 4c). Further, more GFP^hi^ cells had a central memory phenotype compared to GFP^lo^ cells (Fig. 4d). In addition to the strength of signal, it was possible that cells stimulated later in the response would have higher GFP levels and prior data suggested that “late comers” to the immune responses preferentially enter the memory compartment [31, 32]. To test whether the GFP^hi^ cells represented late comer cells or cells with a higher TCR signal strength, we assayed the levels of MHC tetramer staining on GFP^hi^ and GFP^lo^ T cells. When normalized to surface TCRβ levels, the intensity of MHC tetramer stains is directly proportional to TCR affinity [33–36]. Importantly, the MHC tetramer staining intensity of the GFP^hi^ cells was significantly higher than the staining intensity of the GFP^lo^ cells (Fig. 4e). These data are consistent with the concept that TCR signal strength promotes Bim and Nur77 expression and drives the development of pre-memory cells.

### Bcl-2 antagonizes Bim to promote memory T-cell survival

**Fig. 5:**
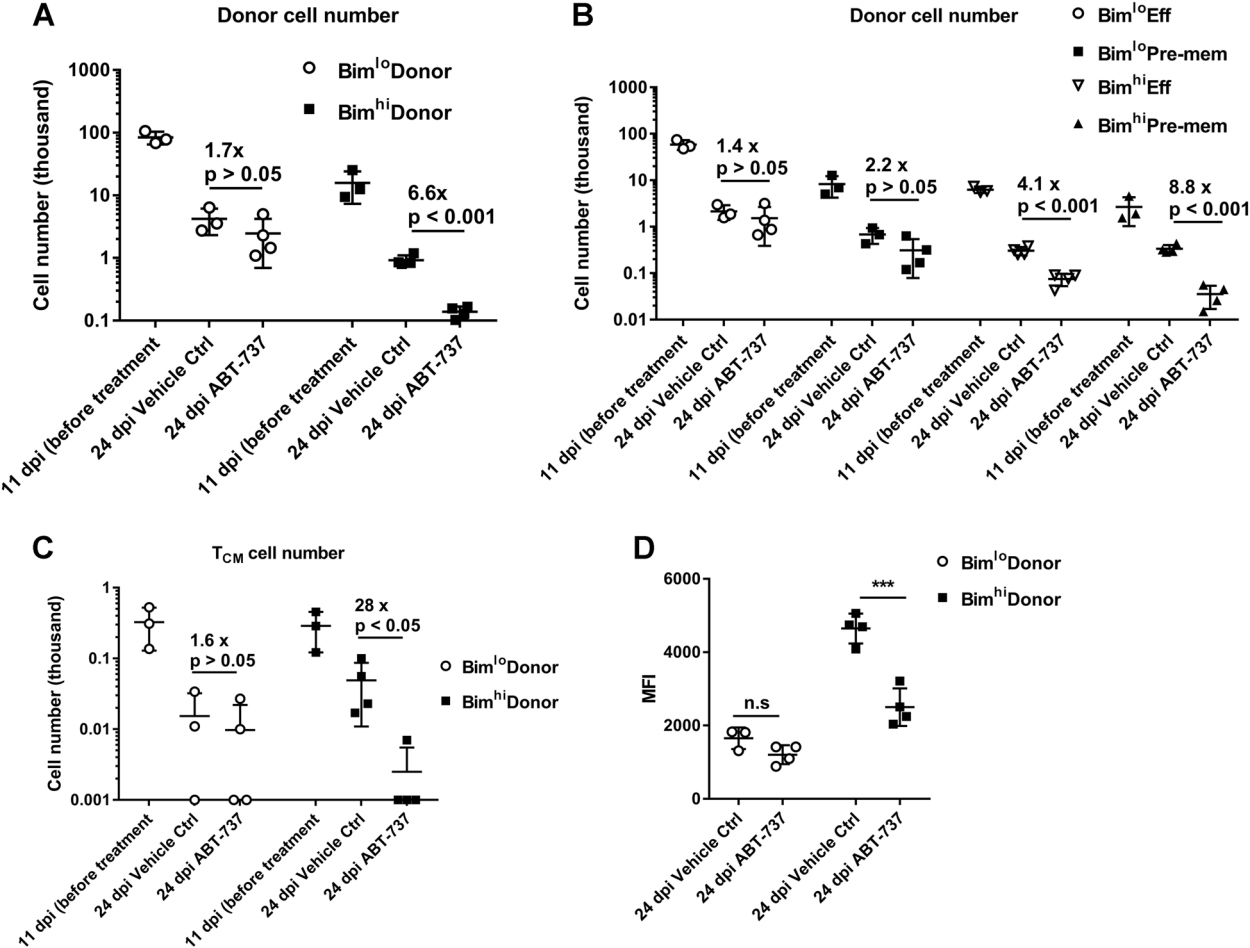
Bcl-2 antagonizes Bim to promote pre-memory cell and T_CM_-cell survival. **a** Total transferred P14-Bim-mCherry reporter cell numbers or **b** transferred terminal effector (Eff) or pre-memory (Pre-mem) cell numbers harvested from one spleen on 11 dpi (1 day after transfer, *n* = 3) or 24 dpi (14 days after transfer), with vehicle or ABT-737 treatment (*n* = 4). Bar graph (mean ± SD) compares the mice treated with vehicle or ABT-737 within the mice receiving Bim-mCherry^hi^ cells or the mice receiving Bim-mCherry^lo^ cells. Cell number fold differences and *p*-value are shown (unpaired two-tailed Student’s *t-*test). **c** Bar graphs (mean ± SD) show T_CM_-cell numbers within the mice receiving Bim^hi^ (black bars) or Bim^lo^ (white bars) P14 cells on 11 or 24 dpi. Cell number fold differences and *p-*value are shown (unpaired two-tailed Student’s *t*-test). **d** Bar graphs (mean ± SD) show Bim-mCherry MFI of transferred cells with vehicle or ABT-737 treatment. (***p < 0.001, unpaired two-tailed Student’s *t*-test)

Our data showed that memory T-cell development is associated with strong TCR stimulation, as well as Bim expression; however, it was unclear how cells with a long-lived fate could avoid Bim-driven death. Our prior data suggested a role for Bcl-2 in antagonizing Bim and maintaining the survival of pre-memory cells [15, 22], although we did not specifically determine if Bcl-2 promoted the survival of Bim^hi^ cells. To test if Bcl-2 promoted the survival of Bim^hi^ vs. Bim^lo^ cells, we repeated the adoptive transfer experiment (Supplementay Fig. 4A) and treated the recipient mice with a Bcl-2/Bcl-xL-specific inhibitor, ABT-737 [22] between day 14 and day 23 post-infection. ABT-737 did not have significant effects on Bim^lo^ CD8^+^ T cells during the contraction phase, while it dramatically diminished the number of Bim^hi^ CD8^+^ T cells, especially the cells with pre-memory phenotype (Fig. 5a, b). Strikingly, T_CM_- cell development from Bim^hi^ precursors was substantially decreased by ABT-737 treatment (Fig. 5c). This requirement for Bcl-2 in antagonizing Bim was further strengthened by the observation that relative to the vehicle control, only cells having significantly decreased levels of Bim survived ABT-737 treatment (Fig. 5d). These results show that the expression of Bim is associated with memory T-cell development, that it is kept in check by Bcl-2, and that this balance is critical for the emergence of T_CM_ cells.

## Discussion

In this study, we developed and characterized an mCherry fluorescent reporter mouse, which faithfully reflects endogenous Bim protein levels. As these mice are a transcriptional reporter, these data strongly suggest that Bim expression is controlled largely at the transcriptional level in T cells. This is consistent with a prior report showing a lack of a T-cell phenotype in mice whose phosphorylation sites on Bim (important for post-translational Bim turnover) are mutated [37]. Instead, we find that steady state expression levels are controlled by DNA methylation and are predictive of memory cell fate. It was surprising that higher levels of Bim were associated with long-lived memory cells as Bim is critical for driving the contraction of most effector T cells [2, 11–15]. One explanation is that pre-memory cells express high levels of anti-apoptotic Bcl-2, which allows pre-memory cells to tolerate higher levels of Bim than terminal effector cells [15]. Indeed, when the intrinsic apoptosis was blocked downstream of Bim, due to the deficiency of Bax and Bak, the levels of Bim and Bcl-2 were uncoupled, allowing the survival of Bim^hi^Bcl-2^lo^ pre-memory cells [15].

However, the uneven Bim levels in terminal effector or pre-memory subsets are not solely caused by apoptosis-mediated selection. Strikingly, even in Bax^-^Bak^-^ mice, pre-memory cells still expressed higher levels of Bim than terminal effector cells [15]. This suggests a non-mutually exclusive hypothesis that the expression levels of Bim are determined early during CD8^+^ T-cell differentiation, rather than solely selected by apoptosis pressure. Here, our data further link these two explanations together. The Bim^hi^/Bim^lo^ adoptive transfer experiment confirmed that high levels of Bim are associated with CD8^+^ T-cell memory development, while the data from ABT-737 treatment showed that Bcl-2 protects the memory precursors from apoptosis mediated by Bim.

We envision two major explanations for why there might be such divergent and programmed expression of Bim. First, high expression of Bim in pre-memory and T_CM_ cells ensures the ability to cull these highly proliferative and long-lived cells to prevent potential malignancies. Second, IL-7 and IL-15 are critical regulators of memory CD8^+^ T-cell homeostasis and promote expression of the major Bim antagonist, Bcl-2 [38]. Thus, the high levels of Bim ensure the homeostasis of the memory compartment proportional to cytokine availability.

Our data are also consistent with a prior study using a Bcl-2 reporter mouse, which showed that adoptive transfer of Bcl-2^hi^ cells preferentially gave rise to memory T cells [39]. The authors also made the surprising observation that CD8^+^ effector T cells expressing the very highest levels of Bcl-2 (top 5% of cells) were actually less efficient at forming memory than cells with slightly lower levels of Bcl-2. Our data would suggest that perhaps these cells represent cells that are tip-toeing the balance of Bcl-2 and Bim, and might be most prone to death, if they couldnot sequester sufficient levels of cytokines to maintain high Bcl-2 expression. Alternatively, it is possible that in the cells expressing the highest levels of Bcl-2, other pro-apoptotic factors (i.e., Puma or Noxa) may be involved in culling these cells [16].

Our previous work showed that CD8^+^ T-cell differentiation and memory generation is regulated by DNA methylation, which initiates its effects early and last long after immune contraction [40]. Here, we observed that the levels of Bim are determined early and mostly maintained across the contraction phase, suggesting that CD8^+^ T-cell intrinsic mechanisms likely dominantly control and maintain the levels of Bim. In addition, we found that Bim expression levels are inversely correlated with DNA methylation at the Bim promoter. Similarly, DNA methylation appears to control Bim in several other cell types, including B lymphoma cells [41–44]. Indeed, treatment with a DNA methylation inhibitor upregulates Bim expression in CD8^+^ T cells. Combined, these data suggest that DNA methylation controls the maintenance of Bim expression levels, as well as memory generation. However, the enzyme catalyzing these modifications seems to be different, as DNA methyltransferase 3a controls memory T-cell differentiation but not Bim expression (Supplementary Fig. 5) [40].

High levels of Bim and Nur77 expression were also associated with T_CM_ differentiation and determined early in the response. These data agree with previous studies, suggesting that CD8^+^ T cells expressing high-affinity TCRs or having strong downstream signaling promote CD8^+^ T-cell memory [45–49]. In contrast, other groups reported that weak TCR stimulation induces better pre-memory or T_CM_ differentiation [4, 50–52]. Here, we isolated the CD8^+^ T cells with strong or weak TCR stimulation from the same donor mice, which avoids the extrinsic effects caused by immune environments of different donors, a caveat of some studies using mice engineered to have lower TCR signal strength [51–54]. Further, we found that Nur77-GFP^hi^ cells had higher staining with MHC tetramers providing further evidence that TCR avidity, rather than temporal differences in TCR stimulation, explain the ability of Nur77-GFP^hi^ cells to promote memory development.

While high levels of Nur77 were correlated with Bim expression and memory generation, Nur77 may be more than just a marker in that it transcriptionally controls Bim expression and other genes of the memory program [55–57]. Indeed, deficiency in *Nr4a1* (the gene encoding Nur77), results in higher expression of interferon regulatory factor 4 (IRF4) and decreased numbers of KLRG1^lo^CD127^hi^ pre-memory cells [58]. During CD8^+^ T-cell activation, IRF4 promotes the expression of several transcription factors, including Blimp-1, T-bet, and Id2, which direct the differentiation to the terminal effector population, but it inhibits Eomes, Bcl-6, and Tcf1 [55, 59–61]. Further, IRF4 directly represses Bim expression [55]. This Nur77/IRF4/Bim circuit may explain why the high levels of Nur77 and Bim at the peak of immune responses are associated with memory fates.

In summary, this Bim-mCherry reporter model provides a useful tool to interrogate Bim expression and its association with memory generation and T_CM_ differentiation fates. Indeed, our data show that high TCR affinity/strong TCR signals positively correlated with Bim expression and memory generation. Further, these TCR signals are likely linked to epigenetic control of Bim expression, which ultimately determine memory T cell fate. Collectively, this information will be useful in manipulating immune responses in the context of vaccination, treatment of infection, or cancer immunotherapy. Beyond CD8^+^ T-cell effector fate, Bim is important for kidney development [56], apoptosis of neurons [57], and is downregulated in multiple tumors [41, 62–64], making these mice broadly useful for understanding the role of Bim in cell fate.

## Methods

### Mice and infection model

Bim-mCherry mice were generated by the Gene Targeted Mouse Service Core at the University of Cincinnati. C57BL/6 mice were purchased from Taconic Farms. Nur77^GFP^ mice (C57BL/6-Tg(Nr4a1-EGFP/cre)820Khog/J) and FlpE mice (B6.Cg-*Pvalb*^*tm2.1(flpe)Hze*^/J) were purchased from The Jackson Laboratory. BoyJ mice (B6.SJL-*Ptprc*^*a*^*Pepc*^*b*^/BoyCrCrl) were purchased from Charles River Laboratories, Inc. dLckCre^+^Dnmt3a^fl/fl^ mice were generated as previously described [40]. CD45.1^+^ P14 TCR transgenic mice were a gift of Dr. M Jordan and were crossed to Bim-mCherry mice. In the infection experiment, mice were infected with 2 × 10^5 ^pfu of LCMV (Armstrong strain). The spleens were harvested on indicated time points. All mice were used between 6 and 12 weeks of age. Animals were housed under specific pathogen-free conditions in the Division of Veterinary Services, and experimental procedures were reviewed and approved by the Institutional Animal Care and Use Committee at the Cincinnati Children’s Hospital Research Foundation.

### Cell processes, cell culture, and flow cytometry

Thymi or spleens from individual mice were harvested and crushed through a 100 μm mesh strainer to generate single-cell suspensions. Splenic CD8^+^ T cells were cultured in S-MEM media (Gibco) containing 10% Fetal Bovine Serum (FBS), with supplement of 20 ng/ml of IL-2 (R&D Systems), and indicated concentration of 5-aza-2’-deoxycytidine (Sigma-Aldrich). The cells were stained with H-2D^b^-GP33 tetramer (NIH Tetramer Core Facility) or Abs against CD4, CD8α, TCRβ (BD Biosciences), CD25, CD44, CD45.2, MHC II (I-A/I-E) (eBioscience), CD45.1, CD62L, KLRG1, CD127 (Biolegend), or Bim (Cell Signaling Technology). Intracellular stains were performed using 0.03% saponin for staining. For detection of Bim, secondary anti-rabbit IgG Ab was used (Life Technologies). These cells were further analyzed on a BD LSR II or BD LSRFortessa flow cytometer and analyzed by FACSDiva software (BD Biosciences) or FlowJo software.

### Cell sorting, adoptive transfer, and ABT-737 treatment

Uninfected C57BL/6 mice received 5000 CD8^+^ T cells, isolated from CD45.1^+^ P14-Bim-mCherry mice with CD8a^+^ T-Cell Isolation Kit (Miltenyi Biotec Inc.), through intravenous (i.v.) adoptive transfer and were infected with LCMV one day later. On day 10 post-infection, splenic CD8^+^ T cells were enriched with CD8a^+^ T-Cell Isolation Kit. Bim-mCherry cells, P14-Bim-mCherry cells, or Nur77-GFP cells were further sorted on BD FACSAria II (BD Biosciences) with the assistance of the Research Flow Cytometry Core at CCHMC. One million of sorted CD8^+^ T cells were i.v. adoptive transferred into timed-infected congenic recipients. After 14 days, the lymphocytes in spleen were analyzed by flow cytometry. ABT-737 was a generous gift from Abbott Laboratories [65] and given to mice as previously described [15, 22, 66], 1 mg/mouse/day between days 14 and 23 post-infection.

### DNA methylation assay

Endogenous CD8^+^ T_CM_ cells (CD8^+^CD44^hi^CD62L^hi^) or T_EM_ cells (CD8^+^CD44^hi^CD62L^lo^) from uninfected C57BL/6 mice were sorted out and genomic DNA was isolated using the AllPrep DNA/RNA Mini Kit (Qiagen). For bisulfite sequencing, genomic DNA was bisulfite converted using EZ DNA Methylation Kit (Zymo Research), and the Bim promoter region (−461/ + 4) was amplified by PCR (primer forward: 5′-GGGGATATAGTAGGTGAAGTTGTTG-3′, reverse: 5′-CTACCAATACTCCCCCATTAACC-3′). The products were purified using QIAquick PCR Purification Kit (Qiagen) and cloned into pGEM-T Easy Vectors (Promega). The plasmid DNA from 20 individual clones were sequenced.

### Statistical analysis

Data were analyzed using GraphPad Prism or Microsoft Excel software. Paired or unpaired Student’s *t-*test, ANOVA, or Pearson correlation coefficient test were used as indicated.

## Supplementary information

Supplemental Figure 1

Supplemental Figure 2

Supplemental Figure 3

Supplemental Figure 4

Supplemental Figure 5

supplementary figure legends
