# Supplementary figures and images for: T-cell receptor signal strength and epigenetic control of Bim predict memory CD8+ T-cell fate
Source: Cell Death Differ. 2019 Sep 26;27(4):1214–24. doi: 10.1038/s41418-019-0410-x (PMC7206134; doi:10.1038/s41418-019-0410-x)

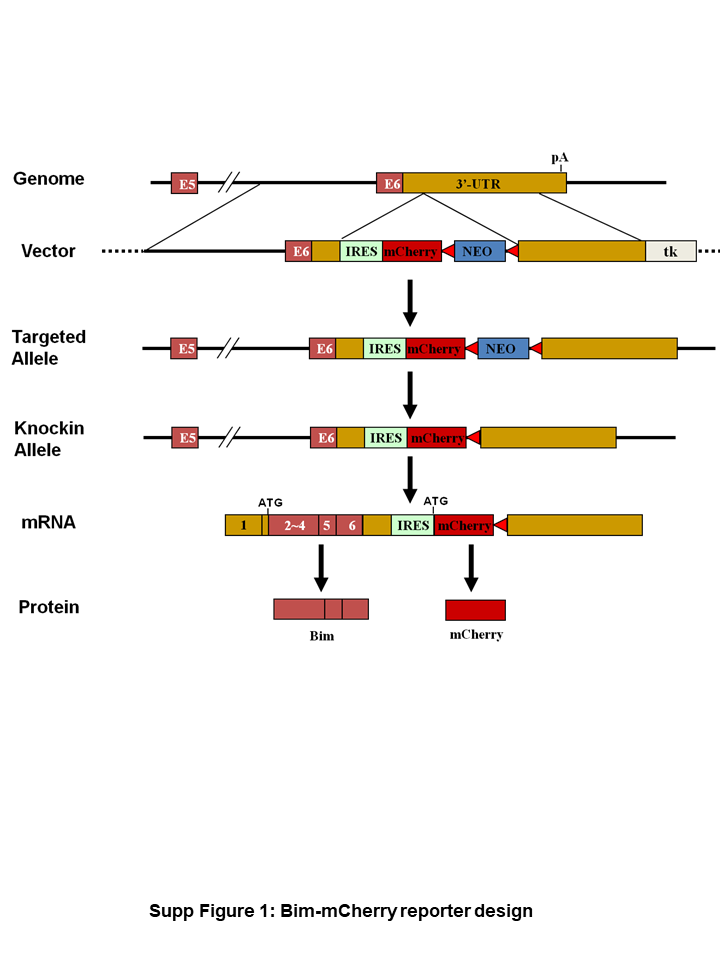

Supplement: Supplementary file 1 — Supplemental Figure 1 [file 41418_2019_410_MOESM1_ESM.tif]

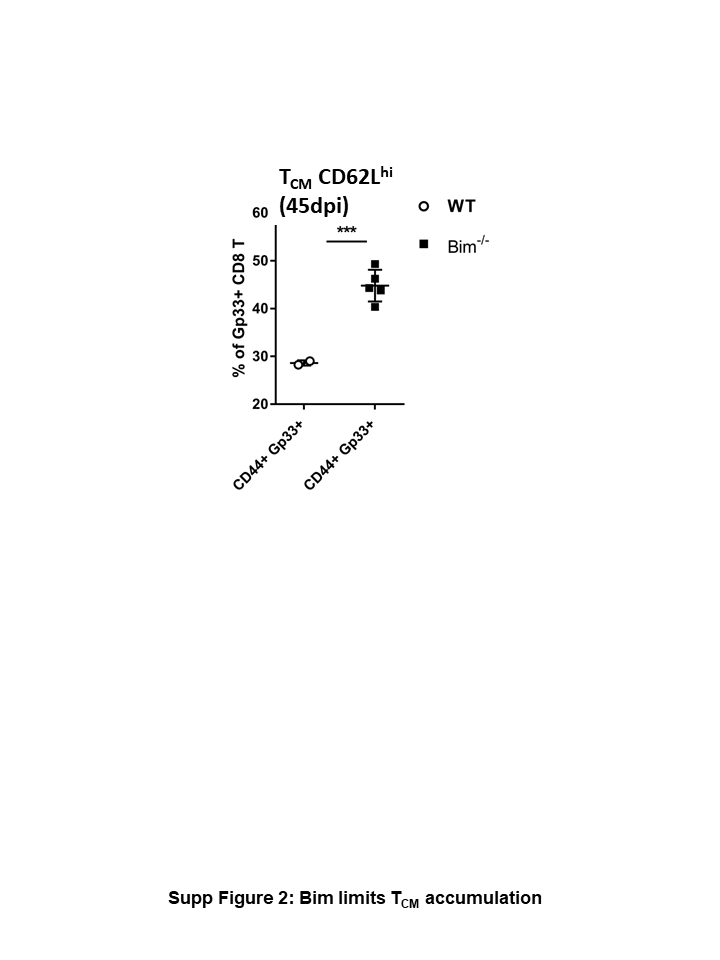

Supplement: Supplementary file 2 — Supplemental Figure 2 [file 41418_2019_410_MOESM2_ESM.tif]

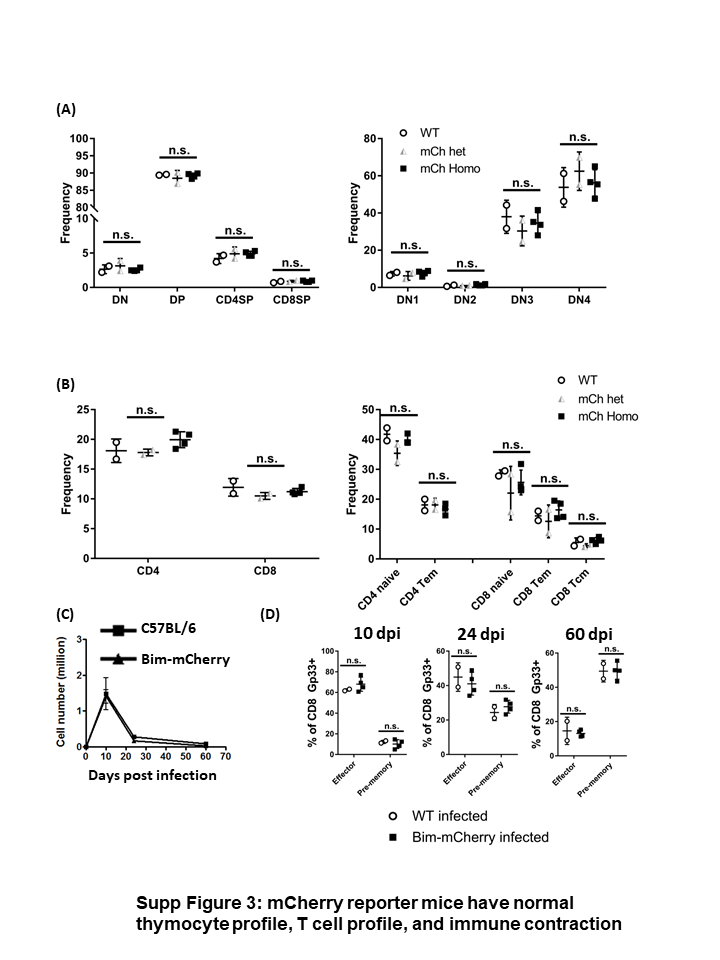

Supplement: Supplementary file 3 — Supplemental Figure 3 [file 41418_2019_410_MOESM3_ESM.tif]

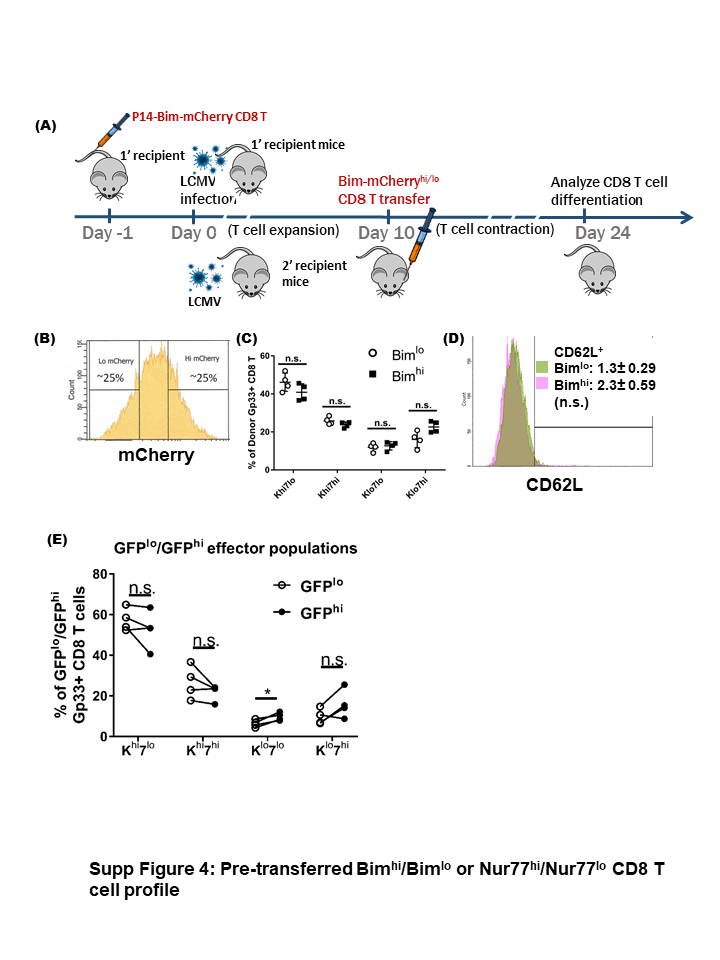

Supplement: Supplementary file 4 — Supplemental Figure 4 [file 41418_2019_410_MOESM4_ESM.tif]

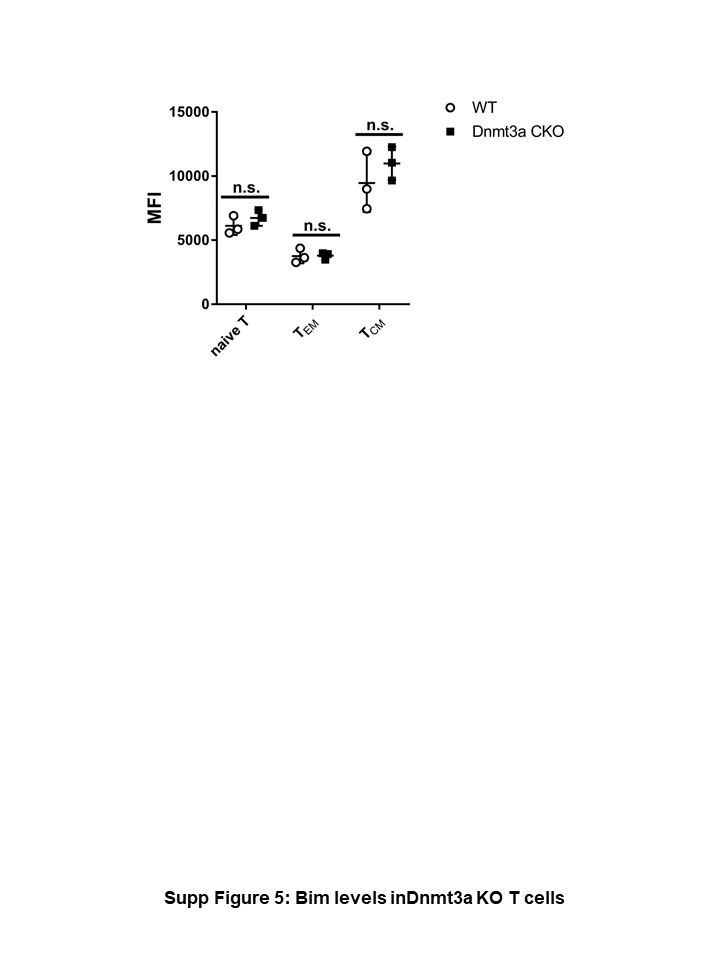

Supplement: Supplementary file 5 — Supplemental Figure 5 [file 41418_2019_410_MOESM5_ESM.tif]
