# Supplementary material for: T-cell receptor signal strength and epigenetic control of Bim predict memory CD8+ T-cell fate
Source: Cell Death Differ. 2019 Sep 26;27(4):1214–24. doi: 10.1038/s41418-019-0410-x (PMC7206134; doi:10.1038/s41418-019-0410-x)
Supplement: Supplementary file 6 — supplementary figure legends [file 41418_2019_410_MOESM6_ESM.docx]

**Supplementary Figure Legends**

**Supplementary Figure 1.** Bim-mCherry reporter design. Design and construction of the Bim-mCherry targeting vector and the subsequent steps to generate mice heterozygous for Bim-mCherry were performed by the Gene Targeted Mouse Service Core at the University of Cincinnati. Briefly, the vector contained an IRES-mCherry cassette flanked with 3.4 kb and 3.0 kb of Bim genomic sequence designed to insert the cassette into the 3’-UTR of *Bcl2l11* (Bim) gene. A neomycin resistance gene (NEO) and a thymidine kinase gene (tk) were included for positive and negative selection, respectively. The construct was electroporated into mouse ES cells derived from a C57B/6 strain, and ES cell clones with correctly targeted Bim-mCherry were identified by PCR and further confirmed by Southern blot analysis. Two correctly targeted ES cell clones were injected into blastocysts to generate chimeras, which were then bred with albino C57B/6 mice to obtain ES cell-derived offspring as determined by the presence of black coat color. Black mice were further analyzed by PCR for germline transmission of the targeted Bim gene. A founder having faithful and bright mCherry expression was chosen and bred to FlpE mice to remove the neomycin cassette and then bred back to C57BL/6 mice for subsequent work.

**Supplementary Figure 2.** Bar graphs (Mean ± SD) show frequencies of LCMV-specific T_CM_ cells from C57BL/6 or Bim^-/-^ mice on 45 dpi (***p<0.001).

**Supplementary Figure 3.** mCherry reporter homozygous (mCh Homo) mice or mCherry reporter heterozygous (mCh het) mice have normal thymocyte profile, T cell profile, and immune contraction. (A) The left bar graph (Mean ± SD) shows frequencies of thymic DN (CD4^-^CD8^-^), DP (CD4^+^CD8^+^), CD4SP (CD4^+^CD8^-^), or CD8SP (CD4^-^CD8^+^) cells. The right bar graph shows frequencies of DN1 (CD44^+^CD25^-^), DN2 (CD44^+^CD25^+^), DN3 (CD44^-^CD25^+^), or DN4 (CD44^-^CD25^-^) cells among DN thymocytes. No difference was observed by ANOVA (n=3). (B) The left bar graph (Mean ± SD) shows frequencies of splenic CD4^+^ T cells (CD4^+^CD8^-^) or CD8^+^ T cells (CD4^-^CD8^+^). The right bar graph shows frequencies of naïve (CD44^lo^CD62L^hi^), T_EM_ (CD44^hi^CD62L^lo^), or T_CM_ (CD44^hi^CD62L^hi^) cells among CD4^+^ or CD8^+^ T cells. No difference was observed by ANOVA (n=3). (C) The same contraction rates of LCMV-specific H-2D^b^-GP33 tetramer^+^ CD8^+^ T cells in C57BL/6 and Bim-mCherry reporter mice were observed after LCMV infection (n=3-5). (D) Frequencies of splenic terminal effector or pre-memory populations among LCMV-specific CD8^+^ T cells are shown (Mean ± SD, n=3-5, unpaired two-tailed Student’s *t* test).

**Supplementary Figure 4.** Transfer strategy and characterization of Bim^hi^ / Bim^lo^ or Nur77^hi^ / Nur77^lo^ CD8^+^ T cells. (A) The infection and adoptive transfer design. (B) Representative gating strategy for Bim^hi^/Bim^lo^ CD8^+^ T cell sorting. (C, D) Phenotype of Bim^hi^ or Bim^lo^ P14 CD8^+^ T cells 10 days after infection. (Mean ± SD, n=4, unpaired two-tailed Student’s *t* test). The experiments were performed four times using P14-Bim-mCherry cells or polyclonal CD8^+^ Bim-mCherry cells with similar results. (E) Phenotype of Nur77^hi^ or Nur77^lo^ LCMV-specific CD8^+^ T cells 10 days after infection. (n=4, paired two-tailed Student’s *t* test). The experiments were performed twice with similar results.

**Supplementary Figure 5.** Bim levels in Dnmt3a KO T cells. Bim expression levels of naïve (CD44^lo^CD62L^hi^), T_EM_ (CD44^hi^CD62L^lo^), or T_CM_ (CD44^hi^CD62L^hi^) CD8^+^ T cells. Bar graph compares C57BL/6 (WT) and dLckCre^+^Dnmt3a^fl/fl^ (Dnmt3a CKO) cells. (Mean ± SD, n=3, unpaired two-tailed Student’s *t* test). The experiments were performed twice with similar results.
